# Epidemiology of *Mycobacterium bovis* Disease in Humans in England, Wales, and Northern Ireland, 2002–2014

**DOI:** 10.3201/eid2303.161408

**Published:** 2017-03

**Authors:** Jennifer A. Davidson, Miranda G. Loutet, Catherine O’Connor, Cathriona Kearns, Robert M.M. Smith, Maeve K. Lalor, H. Lucy Thomas, Ibrahim Abubakar, Dominik Zenner

**Affiliations:** Public Health England, London, UK (J.A. Davidson, M.G. Loutet, C. O’Connor, M.K. Lalor, H.L. Thomas, I. Abubakar, D. Zenner); P; ublic Health Agency Northern Ireland, Belfast, Ireland, UK (C. Kearns);; Public Health Wales, Cardiff, Wales, UK (R.M.M. Smith);; University College London, London (M.K. Lalor, H.L Thomas, I. Abubakar, D. Zenner)

**Keywords:** Mycobacterium bovis, Mycobacterium tuberculosis, tuberculosis and other mycobacteria, zoonoses, zoonotic diseases, epidemiology, England, Wales, Northern Ireland, bacteria

## Abstract

Despite control efforts, *Mycobacterium bovis* incidence among cattle remains high in parts of England, Wales, and Northern Ireland, attracting political and public health interest in potential spread from animals to humans. To determine incidence among humans and to identify associated factors, we conducted a retrospective cohort analysis of human *M. bovis* cases in England, Wales, and Northern Ireland during 2002–2014. We identified 357 cases and observed increased annual case numbers (from 17 to 35) and rates. Most patients were >65 years of age and born in the United Kingdom. The median age of UK-born patients decreased over time. For 74% of patients, exposure to risk factors accounting for *M. bovis* acquisition, most frequently consumption of unpasteurized milk, was known. Despite the small increase in case numbers and reduction in patient age, *M. bovis* infection of humans in England, Wales, and Northern Ireland remains rare.

## Abstract 2

Despite slightly increased cases in these areas, human infection with this cattle pathogen remains rare.

After the 1960s, the number of human cases of tuberculosis (TB) caused by *Mycobacterium bovis* decreased significantly in England, Wales, and Northern Ireland, co-inciding with widespread implementation of milk product pasteurization and national bovine TB control programs (*1**–**3*). During the past 2 decades in these 3 countries, an average of 30 cases of *M. bovis* in humans occurred annually; numbers decreased in the early 2000s before again increasing (*4**–**6*). During the same period, incidence of *M. bovis* in cattle herds in parts of England, Wales, and Northern Ireland increased substantially but has now plateaued (*4**,**7**–**9*).

*M. bovis* control (*2**,**7**,**10**,**11*) attracts political, public health, and media interest because of potential spread from animals to humans, effects on animal health and trade (*1*), and the role of wildlife in the transmission cycle (*12*). Highly visible interventions, including wildlife management to prevent transmission to livestock, are used to attempt to control *M. bovis* spread (*6**,**9**,**10*), thereby protecting human health.

Compared with other countries in western Europe, the rate of TB among humans in the United Kingdom is high: 9.6 cases/100,000 population (6,240 cases) in 2015 (*13*). Most TB cases occurred in those born abroad, who probably acquired infection before entering the United Kingdom. Although only 1.1% (42 cases) of culture-confirmed TB cases were caused by *M. bovis *(*6*), it remains a public health priority.

The drivers of the epidemiology of *M. tuberculosis* are well described (*13**–**15*). However, there is comparatively less information on the sources of *M. bovis* in humans, other than the recognized risks of unpasteurized milk consumption and close contact with infected cattle (*1**,**3*). We provide an update on the demographic characteristics of humans with *M. bovis* disease in England, Wales, and Northern Ireland (*16*). To address the gap in knowledge regarding lesser known sources of acquisition, we describe the demographic and clinical characteristics of humans with TB caused by *M. bovis* compared with *M. tuberculosis*. In addition, we describe potential human exposures that may indicate *M. bovis* acquisition and include a genotyping comparison of the causative organisms.

## Materials and Methods

### Study Population and Definitions

Our retrospective cohort study included all human *M. bovis* patients in the descriptive analysis. To describe demographic and clinical characteristics associated with *M. bovis* disease, we compared all *M. bovis* notified patients with all *M. tuberculosis* notified patients. Potential exposures to risk factors associated with *M. bovis* acquisition were collected through a questionnaire and limited to *M. bovis* cases identified during 2006–2014, when the questionnaire return rate was high (>80%).

An *M. bovis* case was defined as a culture-confirmed human case of TB speciated as *M. bovis* isolated during 2002–2014. A notified *M. bovis* case was an *M. bovis* case clinically notified to the Enhanced TB Surveillance system (ETS); a nonnotified *M. bovis* case was an *M. bovis* not reported clinically to ETS. An *M. tuberculosis* notified case was defined as a culture-confirmed human case of TB speciated as *M. tuberculosis* isolated during 2002–2014 and clinically notified to ETS. 

### Data Collection

Results from culture-positive laboratory isolates were sent from *Mycobacterium* reference laboratories in England, Wales, and Northern Ireland to Public Health England. These results were matched with notified TB cases from ETS, used for statutory notification of TB, by use of a probabilistic matching method (*17*).

Data on demographics (age, sex, ethnicity, country of birth, time since UK entry, address, and occupation); clinical factors (site of disease and previous diagnosis); and social risk factors (current or past imprisonment, homelessness, drug and alcohol misuse) were obtained from ETS notifications. For nonnotified *M. bovis* cases, the only patient demographic information available was age, sex, and address; the disease site was inferred from specimen site. For analysis, we used the age groups 0–14, 15–44, 45–64, and >65 years and the ethnic groups white, black African, Indian subcontinent (Indian, Pakistani, and Bangladeshi grouped together), and other. After assignment to a geographic area of residence based on address, the place of residence was classified as rural or urban by using 2011 census classifications (*18*).

After identification of an *M. bovis* case (based on phenotypic, PCR, and genotypic methods [*19**,**20*]), a questionnaire (Technical Appendix) (*21*) was issued to collect information on potential recognized current or past *M. bovis* exposures. These exposures were contact with a human TB patient, travel (for >2 weeks) to or residence in a country with high TB incidence (defined as having an estimated rate of >40 cases/100,000 population during 2002–2014), consumption of unpasteurized milk product, occupational contact with animals, physical contact with wild (nondomestic) animals, and physical contact with any animal with TB (including pets).

### *M. bovis* Trend Analysis

We calculated incidence rates per 100,000 population by using mid-year population estimates produced by the UK Office for National Statistics (*22*). We used Poisson regression to calculate the incidence rate ratio to assess the trend in *M. bovis* incidence over time. We used a nonparametric test for trend across ordered groups to assess the age trend of *M. bovis* patients and the χ^2^ test for trend to assess the proportion of *M. bovis* among culture-confirmed TB cases.

### Factors Associated with *M. bovis* Disease and *M. tuberculosis* Disease

Demographic and clinical characteristics for *M. bovis* notified patients were compared with those of *M. tuberculosis* notified patients by using univariable and multivariable logistic regression to calculate odds ratios to identify factors associated with *M. bovis* disease. A forward stepwise multivariable logistic regression model was used, including sex and all variables with a p value <0.2 in univariable analysis; likelihood ratios were assessed after each stepwise addition to the model. In addition, we conducted a stratified analysis based on place of birth (UK-born/non–UK-born). A p value of <0.05 was considered statistically significant. We tested interactions between biologically and statistically plausible variables in the model by using likelihood ratios. All analyses were conducted by using Stata 13.1 (StataCorp LLC, College Station, TX, USA).

### Exposures to Risk Factors Associated with *M. bovis* Disease

To identify frequent exposure to risk factors among the cohort, we used case exposure history, as collected through the questionnaire (Technical Appendix), for descriptive analysis. In addition to obtaining questionnaire information about contact with another human TB patient, for culture-positive isolates identified during 2010–2014, we also obtained 24-loci mycobacterial interspersed repetitive unit–variable tandem repeat (MIRU-VNTR) strain typing results (*20*) from *Mycobacterium* reference laboratories. This information enabled us to identify strain type clusters, defined as >2 human TB cases with indistinguishable MIRU-VNTR profiles (or with an indistinguishable profiles but with 1 case only typed to 23 loci), Clustered cases were further investigated to identify possible epidemiologic links, the identification of which suggest recent human-to-human transmission (*23*).

## Results

### Demographics of *M. bovis* Patients

**Figure 1:**
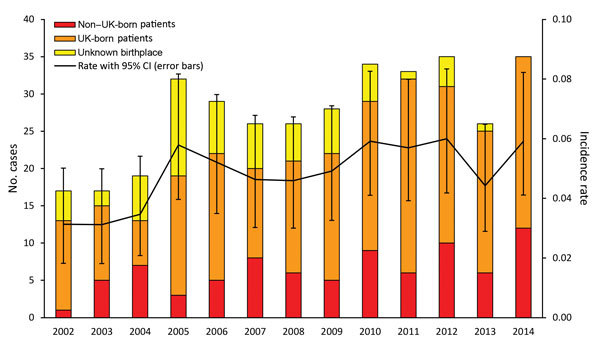
Annual number and incidence rate (no. cases/100,000 population) of notified *Mycobacterium bovis* cases by patient place of birth, England, Wales, and Northern Ireland, 2002–2014. Unknown place of birth includes notifications with an unknown place of birth and cases that have not been notified.

For 2002–2014, we identified 357 culture-confirmed cases of *M. bovis* disease in humans. During this time, the proportion of all culture-confirmed TB cases speciated as *M. bovis* increased from 0.4% to 0.9% (p<0.001). Annual case numbers ranged from 17 in 2002 to 35 in 2014, and the incidence rate fluctuated between 0.03 and 0.06 cases/100,000 population (Figure 1); the incidence rate ratio per year was 1.04 (95% CI 1.01–1.07). Overall, 92.2% (329/357) of *M. bovis* cases were notified to ETS; since 2011, all identified cases have been notified.

**Figure 2:**
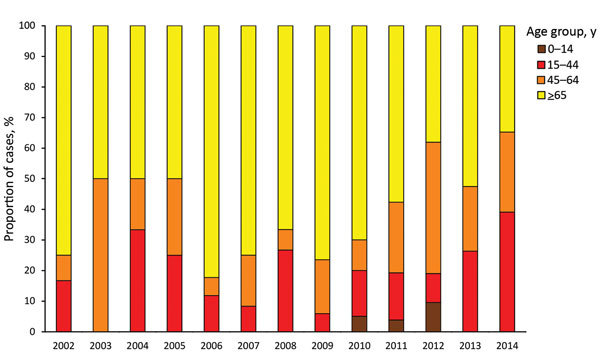
Annual number of notified UK-born *Mycobacterium bovis* cases, by patient age group, England, Wales, and Northern Ireland, 2002–2014.

Among 297 *M. bovis* patients for whom place of birth was recorded, 214 (72.1%) were born in the United Kingdom. The most frequent countries of birth for the others were Nigeria (18 patients), Morocco (9 patients), and India (8 patients). The age distribution differed significantly between those born and not born in the United Kingdom (p<0.001) (Table 1). The median age of UK-born patients fluctuated over time, from 71 years (interquartile range 60–76) in 2002 to 53 years (interquartile range 35–79) in 2014 (p = 0.099), as did the proportion of cases by age group (Figure 2). Only 6 *M. bovis* cases in patients <15 years of age were reported (all 11–14 years of age).

**Table 1:** Characteristics of patients with *Mycobacterium bovis *disease, England, Wales, and Northern Ireland, 2002–2014*

Characteristic†	All patients, no. (%), n = 357‡	UK-born patients, no. (%), n = 214§	Non–UK-born patients, no. (%), n = 83¶
Age group, y			
0–14	6 (1.7)	4 (1.9)	2 (2.4)
15–44	106 (29.7)	39 (18.2)	54 (65.1)
45–64	70 (19.6)	45 (21.0)	12 (14.5)
>65	175 (49.0)	126 (58.9)	15 (18.1)
Male sex	196 (55.1)	130 (60.8)	37 (44.6)
Ethnicity			
White	230 (73.0)	199 (93.9)	15 (18.5)
Black African	37 (11.8)	2 (0.9)	35 (43.2)
Indian subcontinent	16 (5.1)	3 (1.4)	9 (11.1)
Other	32 (10.2)	8 (3.8)	22 (27.2)
Time since entered United Kingdom, y			
<2	NA	NA	10 (14.7)
2–5	NA	NA	17 (25.0)
6–10	NA	NA	20 (29.4)
>10	NA	NA	21 (30.9)
Place of residence			
Rural	86 (24.9)	62 (29.0)	9 (10.8)
Urban	259 (75.1)	152 (71.0)	74 (89.2)
Pulmonary TB#			
Yes	199 (56.9)	131 (61.5)	38 (45.8)
No	151 (42.3)	82 (38.5)	45 (54.2)
>1 social risk factor**	12 (7.9)	6 (5.9)	5 (11.1)
Previous TB diagnosis	17 (6.1)	13 (6.7)	5 (5.4)

*IQR, interquartile range; NA, not applicable; TB, tuberculosis.  †Sex, age, and site of disease reported for all cases (excluding breakdowns by birth in or not in the United Kingdom); all other characteristics reported only for notified cases. ‡Median age (IQR) 58 (36–77) y. §Median age (IQR) 70 (52–79) y. ¶Median age (IQR) 35 (28–58) y. #Pulmonary TB with or without extrapulmonary TB, those recorded as “no” had exclusively extrapulmonary TB. **Data only available from 2010 on.

**Figure 3:**
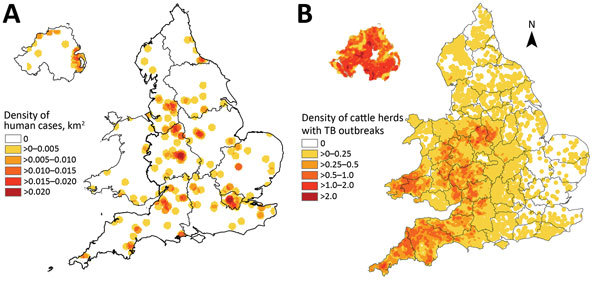
Cases of *Mycobacterium bovis* disease in England, Wales, and Northern Ireland, 2002–2014. A) Density of human cases. B) Density of cattle herds with TB outbreaks. This material is based on Crown Copyright and is reproduced with the permission of Land & Property Services under delegated authority from the Controller of Her Majesty’s Stationery Office.

For all 3 countries, the highest proportion of *M. bovis* patients resided in London, England (18.5%; 66/357), followed by the South West (15.4%; 55) and West Midlands regions of England (13.2%; 47) (Figure 3, panel A). However, the incidence rate was highest in Northern Ireland (0.11 cases/100,000 population), followed by the South West (0.08/100,000) and West Midlands (0.07/100,000) regions. The highest proportion (71.9%; 41/57) of *M. bovis* patients not born in the United Kingdom lived in London. In comparison, 85.1% (40/47) and 82.1% (32/39) of patients from the South West and West Midlands, respectively, were born in the United Kingdom.

### Comparison between Notified *M. bovis* and *M. tuberculosis* Patients

Univariable analysis showed that, when compared with *M. tuberculosis* notified patients, *M. bovis* notified patients were more likely to be >45 years of age, born in the United Kingdom, of an ethnic group other than that of the Indian subcontinent, live in a rural area, and work in agricultural or animal-related occupations. *M. bovis* patients were less likely than *M. tuberculosis* patients to have pulmonary disease. Multivariable analysis showed that the same factors, other than age, were independently associated with *M. bovis*; only those >65 years of age were more likely to have *M. bovis *disease. The strongest risk factor for *M. bovis* disease was working in an agricultural or animal-related occupation (adjusted odds ratio 29.5, 95% CI 16.9–51.6; Table 2). The model showed no interactions between explanatory variables. Analysis stratifying by place of birth (UK-born vs. non–UK-born) indicated that the same variables were significant.

**Table 2:** Demographics and risk factors for patients with *Mycobacterium tuberculosis* and *M. bovis *disease, England, Wales, and Northern Ireland, 2002–2014*

Characteristic	*M. bovis* patients, no. (%), n = 329	*M. tuberculosis* patients, no. (%), n = 58,540	Univariable analysis			Multivariable analysis	
			OR (95% CI)	p value		OR (95% CI)	p value
Age group, y							
0–14	6 (1.8)	1,200 (2.1)	1.9 (0.8–4.3)	<0.001		1.5 (0.6–3.6)	<0.001
15–44	102 (31.0)	38,558 (65.9)	Referent			Referent	
45–64	61 (18.5)	10,953 (18.7)	2.1 (1.5–2.9)			1.3 (0.9–2.0)	
>65	160 (48.6)	7,826 (13.4)	7.7 (6.0–9.9)			3.6 (2.6–5.2)	
Sex							
M	179 (54.4)	33,715 (57.7)	0.9 (0.7–1.1)	0.229		0.9 (0.7–1.1)	0.287
F	150 (45.6)	24,721 (42.3)	Referent			Referent	
UK-born							
Yes	214 (72.1)	13,576 (24.7)	7.9 (6.1–10.1)	<0.001		2.6 (1.7–4.1)	<0.001
No	83 (27.9)	71,361 (75.3)	Referent			Referent	
Ethnicity							
White	230 (73)	11,968 (21.1)	28.0 (16.8–46.4)	<0.001		14.6 (7.2–26.9)	<0.001
Black African	37 (11.8)	12,501 (22.1)	4.3 (2.4–7.7)			7.4 (3.7–14.9)	
Indian subcontinent†	16 (5.1)	23,285 (41.1)	Referent			Referent	
Other	32 (10.2)	8,905 (15.7)	5.2 (2.9–9.5)			7.3 (3.6–14.8)	
Occupation							
Agricultural/animal contact work	20 (7.9)	116 (0.3)	32.4 (19.8–53.0)	<0.001		29.5 (16.9–51.6)	<0.001
Other	232 (92.1)	43,698 (99.7)	Referent			Referent	
Site of disease							
Pulmonary	193 (58.8)	37,580 (64.2)	0.8 (0.6–1.0)	0.048		0.4 (0.3–0.5)	<0.001
Extrapulmonary only	135 (41.2)	20,938 (35.8)	Referent			Referent	
Place of residence							
Rural	81 (24.6)	1,944 (3.3)	9.5 (7.3–12.2)	<0.001		2.8 (2.0–3.9)	<0.001
Urban	248 (75.4)	56,380 (96.7)	Referent			Referent	

*Interactions between 1) place of birth (UK-born/non–UK-born) and all of the other variables (age, sex, ethnicity, occupation, site of disease, place of residence [rural/urban]) and 2) age and site of disease or place of residence (rural/urban) were tested. No significant interactions existed in the model. OR, odds ratio. †Indian, Bangladeshi, and Pakistani ethnic groups.

### *M. bovis* Patient Exposure to Risk Factors

Of the 272 *M. bovis* patients identified during 2006–2014, exposure questionnaires were completed for 241 (88.6%). Of these, 179 (74.3%) reported exposure to at least 1 risk factor for *M. bovis* acquisition; 78 (43.6%) reported 1 exposure, 57 (31.8%) 2 exposures, 28 (15.6%) 3 exposures, and 16 (8.9%) 4 exposures. For 6 patients, no exposure was known; for the remaining 56 patients, data were missing for >1 risk factor and the patients could not be classified as not having been exposed to a risk factor.

The most frequently reported exposure was consumption of unpasteurized milk products (65.7%, 109/166; Table 3); proportions reporting this factor were similar among those born in the United Kingdom and those born elsewhere. Among those for whom the most recent consumption of unpasteurized milk product was known, most (85.9%, 55/64) had consumed the product >5 years before TB diagnosis; 42.2% (27/64) were >50 years of age before diagnosis. No change in the age distribution of patients consuming unpasteurized milk was identified over time; most (56.0%, 61/109) were >65 years of age.

**Table 3:** Risk factor exposures reported by patients with *Mycobacterium bovis* disease, England, Wales, and Northern Ireland, 2006–2014*

Exposure	No. characteristics/no. with information recorded (%)		
	All patients	UK-born patients	Non–UK-born patients
Consumption of unpasteurized milk products	109/166 (65.7)	74/112 (66.1)	25/37 (67.6)
In United Kingdom	66/88 (75.0)	60/64 (93.8)	1/15 (6.7)
Travel or residence in a high incidence country	77/203 (37.9)	29/126 (23.0)	44/56 (78.6)
Work related animal exposure	51/103 (49.5)	35/71 (49.3)	7/22 (31.8)
In United Kingdom	33/37 (89.2)	28/29 (96.6)	2/4 (50.0)
Contact with human patient with TB	33/181 (18.2)	21/120 (17.5)	10/42 (23.8)
In United Kingdom	36/38 (94.7)	19/19 (100)	6/8 (75.0)
Physical contact with wild animal	18/126 (14.3)	15/92 (16.3)	0/25 (0)
In United Kingdom	10/13 (76.9)	7/10 (70.0)	Not applicable
Physical contact with animal with TB	18/99 (18.2)	14/76 (18.4)	2/18 (11.1)
In United Kingdom	10/1 (90.9)	9/10 (90.0)	1/1 (100)
Pet with TB†	2	2	0
Farm animal with TB†	11	9	1
No exposure†	6	6	0

*TB, tuberculosis. †Denominator not available.

Contact with a human TB patient was reported by 18.2% (33/181), but for most, recorded information was insufficient to identify the contact, particularly if the contact was not recent. Where known, 80.8% (21/26) of contacts occurred >5 years before TB diagnosis. From 24-loci MIRU-VNTR strain typing data available during 2010–2014, a total of 48.7% (57/117) of patients (of which 46 were born in the United Kingdom and 9 were not) were in 15 *M. bovis* strain type clusters. One cluster contained exclusively patients not born in the United Kingdom and 7 exclusively born in the United Kingdom; 2 of the latter clusters contained the only epidemiologically linked human patients, each with a pair of household contacts.

Recent acquisition of infection cannot be directly measured, but the rate of *M. bovis* disease among children, along with their exposures, can provide an indirect indicator of recent acquisition. Exposure information was available for 5/6 *M. bovis* patients <15 years of age and suggested potential overseas acquisition; 5 had traveled to a country where TB incidence was high, 1 of whom had consumed unpasteurized milk while abroad. For 1 child not born in the United Kingdom, a questionnaire response was not obtained.

Overall, among those for whom location of exposure was known, 59.1% (97/164) of patients were exposed to >1 risk factor in the United Kingdom (Table 3). Among those not born in the United Kingdom, 18.0% (9/50) were known to have been exposed to a risk factor while in the United Kingdom, but 4 of the 9 also were exposed outside the United Kingdom.

## Discussion

Our findings confirm that *M. bovis* disease remains rare among humans in England, Wales, and Northern Ireland. Over the study period, the annual rate of *M. bovis* disease and the proportion of culture-confirmed TB cases with *M. bovis* identified as the cause displayed a small but statistically significant increase; annual case numbers for the past 10 years were similar to those for the early 1990s (*4*). Although speciation has improved from the use of strain typing results (*19**,**20*), this improvement is unlikely to account for all of the increase identified. Although the previous study by Jalava et al. (*4*) and our study overlap by 2 years, our results benefit from improved matching (*17*) between case notification and culture results from 2002 on, thereby providing improved accuracy for reporting annual case numbers.

We identified, unlike previous studies (*4**,**5*), that although the number of *M. bovis* patients not born in the United Kingdom remained low and fluctuated over time, the annual number of cases in this group increased slightly over time. Our finding may be confounded by better recording of place of birth but is not unexpected given the increase during this period in the overall number of TB patients not born in the United Kingdom (*13*). Similar to previous findings (*5*), our findings indicate that most *M. bovis* patients not born in the United Kingdom lived in urban areas, specifically London. These patients originated mostly from low-income countries where TB incidence is high and therefore are at higher risk for human-to-human transmission and animal-to-human transmission because of limited detection of *M. bovis* in animals and less frequent milk pasteurization (*24*). Thus, infection was probably acquired before arrival in the United Kingdom and less likely to be related to exposure to risk factors while in the United Kingdom. Unfortunately, speciation is not routinely conducted in many high TB burden, low-income countries (*16*), so it is difficult to identify in which countries incidence of *M. bovis* is high and whether the trends in country of birth for *M. bovis* patients not born in the United Kingdom reflect the global incidence of the disease (*24**,**25*)*.*

We identified a decrease over time in the proportion of UK-born patients >65 years of age and a decrease in the median age, although neither was statistically significant. Previously, most cases in UK-born patients were the result of reactivation of infection acquired before the large rollout of pasteurization (by the 1960s) (*4*), when *M. bovis* incidence was higher. Given the length of time that widespread pasteurization has been in place, progressively fewer cases among the older population are expected as the cohort exposed before pasteurization decreases. It was unexpected that, despite this decrease, the number of *M. bovis* cases occurring in the UK-born population did not reduce over the study period. Instead, the number and proportion of younger UK-born patients increased slightly. Numbers remain small, and it is not possible to yet detect any change in exposures; however, in recent years, the media have reported increased public demand for unpasteurized milk, which, if contaminated, could result in more human infections.

No data are available to quantify unpasteurized milk production or consumption within England, Wales, and Northern Ireland. However, results from a 2012 survey of adult consumer attitudes about unpasteurized milk (*26*) showed that 33% of respondents had consumed unpasteurized milk but only 3% currently consumed unpasteurized milk. Although the proportion who had ever consumed unpasteurized milk was highest among older age groups (18–24 years, 31%; 25–44 years, 28%; 45–64 years, 38%; >65 years, 40%), the proportion of current consumers was higher among younger age groups (18–24 years, 7%; 25–44 years, 4%; 45–64, 1%; >65, 1%). It is possible that increased consumption of unpasteurized milk, as reported by the media, is contributing to the small increase in *M. bovis* cases and may contribute to a change in demographics of patients over time. Although we do not have evidence to confirm, this hypothesis could be explored further through a formal observation study. The time between unpasteurized milk consumption and onset of TB disease among the *M. bovis* patients in our cohort emphasizes that the effects of current unpasteurized milk consumption may not be observed for many years.

The results of combining routine 24-loci MIRU-VNTR typing of *M. bovis* from humans with epidemiologic data provide evidence of only occasional human-to-human *M. bovis* transmission; despite extensive follow-up of the 57 clustered cases, only 2 instances of 2 cases being epidemiologically linked were found. Only 1 prior occurrence of MIRU-VNTR–confirmed (using 15-loci typing) human-to-human transmission of *M. bovis* in the United Kingdom has been documented (*5**,**27*); it occurred before the rollout of routine prospective 24-loci MIRU-VNTR typing. There are also few examples of human-to-human *M. bovis* transmission in countries other than those included in this study (*28**,**29*), suggesting that such transmission is rarely identified. Overall, the proportion of clustering among *M. bovis* cases (49%) was slightly lower than that of the overall proportion among all TB cases (56%) observed in England (*13*).

This analysis also presents findings consistent with those previously reported (*4*). Although the proportion of cases among the older UK-born population seems to be decreasing, over the study period this group accounted for most cases. Our comparative analysis confirmed that the demographic profile of *M. bovis* patients differs from that of *M. tuberculosis* patients. The consumption of unpasteurized milk remained the most frequently reported exposure, and *M. bovis* patients were more likely than *M. tuberculosis* patients to work or have worked in agricultural and animal-related occupations. These findings are reassuring and show that *M. bovis* disease is still largely limited to those with recognized risk factors for infection. Few incidents involving animal-to-human transmission on farms (*1**,**30**,**31*) and a single incident of *M. bovis* transmission from a pet to its owners *32**,**33*) have occurred during the study period. Most animal-to-human transmission remains sporadic, and implementation of additional specific interventions beyond those currently in place (*1**,**2*) would be difficult.

A high proportion of UK-born patients lived in rural areas, especially across the South West and Midlands of England, where *M. bovis* incidence among cattle is high (Figure 3, panel B). Most of these patients reported consumption of unpasteurized milk or contact with animals. However, human patients without such exposures and who reside in these areas where *M. bovis* cattle incidence is high should continue to be monitored and thoroughly investigated to ensure that lesser known exposures are not missed.

Similar to our study, a study in the Netherlands identified that the highest proportion of *M. bovis* cases occurred in the older native population (50%), followed by the foreign-born population (40%) (*34*). In comparison with our study, studies from the United States found that being foreign born (in particular, being of Hispanic ethnicity) and younger were independently associated with *M. bovis *when compared with *M. tuberculosis* (*35**,**36*). The difference in demographic characteristics of *M. bovis* patients in the United States and in England, Wales, and Northern Ireland may be explained by the fact that *M. bovis* in cattle or wildlife is not frequently reported in the United States (*37**,**38*) but is more common in neighboring Mexico (*39**,**40*). Thus, the epidemiology of human *M. bovis* in England, Wales, and Northern Ireland continues to be driven by the past and, to some extent, present prevalence of disease in cattle. Given advances in molecular techniques, improved understanding of animal-to-human transmission will require linking the genotyping results from animals with *M. bovis* infection in England, Wales, and Northern Ireland with data from humans.

Globally, zoonotic TB should be tackled, and the needs of those affected by *M. bovis* disease, namely those in animal-related occupations and those consuming unpasteurized milk from infected animals, should be addressed. The implementation of methods to identify *M. bovis* where culture is not possible have been highlighted as essential (*16**,**41**,**42*). Although findings from England, Wales, and Northern Ireland cannot be extrapolated even to other high-income countries, much less to high TB burden, low-income countries, our study does illustrate the value of monitoring *M. bovis* disease and the data required to do so.

Our study does have some limitations. The exposure questionnaires return rate was 89%, and some responses were missing, which could lead to some error in the estimation of exposures; in addition, nonresponders were more likely to be urban dwellers. Our comparison of *M. bovis* and *M. tuberculosis* patients was limited because exposure questionnaire information was only collected for *M. bovis* patients; therefore, animal-related exposures, travel to countries with high TB incidence, and contact with human TB patients could not be included in the analysis. In addition, patients not born in the United Kingdom, most of whom belong to Indian subcontinent ethnic groups, are more likely missed in analysis because a higher proportion have exclusively extrapulmonary disease (*43*), for which culture confirmation is lower. Approximately 60% of TB cases in England, Wales, and Northern Ireland are culture confirmed; therefore, the estimated *M. bovis* incidence presented in this article is probably an underestimate. The proportion of TB cases culture confirmed over time has remained relatively stable (*44*), so underascertainment should not affect changes in the number or proportion of TB cases caused by *M. bovis*.

In conclusion, we found that *M. bovis* disease continues to account for a small number and low proportion of total TB cases in England, Wales, and Northern Ireland. The proportion of culture-confirmed TB cases caused by *M. bovis* has increased slightly, and the age of UK-born patients has decreased. The reasons are not fully understood, and trends should continue to be monitored. For most patients, exposure to risk factors for *M. bovis* acquisition (e.g., unpasteurized milk consumption, farm work, or contact with a human TB patient) were known. The current control measures in place to prevent animal-to-human spread seem to be effective; such spread occurs in a few isolated incidents and sporadic events. However, to increase understanding of *M. bovis* transmission in England, Wales, and Northern Ireland, we recommend strengthening collaboration between animal and human health, including linking genotyping results.

Technical Appendix.* Mycobacterium bovis* questionnaire for England, Wales, and Northern Ireland.
