# Supplementary material for: Epidemiology of Mycobacterium bovis Disease in Humans in England, Wales, and Northern Ireland, 2002–2014
Source: Emerg Infect Dis. 2017 Mar;23(3):377–86. doi: 10.3201/eid2303.161408 (PMC5382737; doi:10.3201/eid2303.161408)
Supplement: Supplementary file 1 — Technical Appendix. Mycobacterium bovis questionnaire for England, Wales, and Northern Ireland. [file 16-1408-Techapp-s1.pdf]

Article DOI: http://dx.doi.org/10.3201/eid2303.161408

Epidemiology of *Mycobacterium bovis* Disease in Humans in England, Wales, and Northern Ireland, 2002–2014

Technical Appendix

Mycobacterium bovis questionnaire, England, Wales, and Northern Ireland

The questionnaire was introduced in 1993 to collect basis demographic, clinical and exposure information for *M. bovis* cases before the current Enhanced Tuberculosis Surveillance system (ETS) was implemented to collect demographic and clinical data for all TB cases in 1999.

Since this time the questionnaire has undergone slight revisions. In 2004, travel history was added to the questionnaire. In 2012, much of the demographic and clinical information was removed as this was by then available through ETS. Additionally, tickbox options were added to categorise the setting in which contact with a human case of TB occurred. In 2015, the collection of details on unpasterised milk product consumption were expanded to include the type of product (milk, cheese, yogurt) and the animal which the product came from. Addtionally, a tickbox to specific if the patient was a livestock farmer was added (previously only worked in an abattoir, as a vet, with animals in another capacity was collected) and a question to ask about contact with farm animals.

The current exposure data collected are as follows:

CASE BACKGROUND INFORMATION

Please provide details of; if the patient is immunosuppressed, or on immunosuppressive therapy:

Please provide any other relevant clinical details:

CONTACTS OF OTHER HUMAN CASES (DETAILS)

Has the patient ever spent any time in contact with any known (human) cases of tuberculosis?

Yes ☐ No ☐ Unknown ☐ If yes, ETS/LTBR ID: ____________

If yes, please provide details:

Date	Exposure setting		Details
	☐ Household		
	☐ Health care	☐Hospital ☐ Other	
	☐ Education	☐Nursery ☐Primary ☐Secondary ☐Tertiary	
	☐ Detention	☐Prison ☐ Immigration	
	☐ Homeless Hostel	☐Residential Hostel ☐Night shelter ☐Other	
	☐ Other congregate settings	☐Elderly residential ☐Nursing home ☐Pub ☐ Workplace ☐ Other	
	☐ Travel	☐Air ☐Bus ☐Train ☐ Ship	

UNPASTEURISED MILK PRODUCT CONSUMPTION

Has the patient ever consumed unpasteurised milk products? Yes ☐ No ☐ Unknown ☐

If yes, please provide details: Milk ☐ Cheese ☐ Yogurt ☐

Which animal(s) was milk product obtained from: ____________

With what frequency? Once ☐ Occasionally ☐ Often/regularly ☐ Most recent year of consumption: ____________

Where did the patient obtain the unpasteurised milk products? UK ☐ Outside of UK ☐

If in the UK: Own farm ☐ Local farm ☐ Other ☐ And please specify the county: ____________

If outside UK, please specify the country: ____________

Other details ____________

TRAVEL HISTORY

Has the patient travelled or lived outside the UK for more than one month? Yes ☐ No ☐ Unknown ☐
(to a country with high TB prevalence where human-to-human *M.bovis* transmission may occur i.e. the Indian Subcontinent or Sub-Saharan Africa)

If yes, please provide details:

Country	Year of last visit

OCCUPATIONAL DETAILS / ANIMAL CONTACT

Has the patient ever worked: *(include voluntary work, e.g. for animal welfare charities)* - tick all relevant

in an abattoir ☐ as, or with a vet ☐ as, or with a livestock farmer ☐ with animals in another capacity ☐

Has the patient ever: - tick all relevant

Had physical contact with wild animals: Yes ☐ No ☐ Unknown ☐

Had physical contact with farm animals: Yes ☐ No ☐ Unknown ☐

Had other relevant contact (excluding pets) with an animal: Yes ☐ No ☐ Unknown ☐

FURTHER DETAILS OF OCCUPATIONAL / ANIMAL EXPOSURE

If YES to any of the above please provide details:

Animal	Year of most recent contact	Duration (years)	Country (or if in UK county)	Details

CONTACT WITH ANIMALS WITH TB

Has the patient ever: had a pet ☐ Did the pet have: suspected TB ☐ confirmed TB ☐

Has the patient had any contact with farm animals/wild animals with a positive TB test?

Yes ☐ No ☐ Unknown ☐ If yes (or pet had TB), please provide details:

Animal	Year of most recent contact	Duration (years)	Country (or if in UK county)	Details

If the patient has had contact with TB positive farm animals,

Were visible lesions present in carcass? Yes ☐ No ☐ Unknown ☐

Were udder lesions present? Yes ☐ No ☐ Unknown ☐

Herd type: ____________________ Herd size: ________

Please provide any **other information** you think may be relevant to this illness:
